# Exenatide infusion decreases atrial natriuretic peptide levels by reducing cardiac filling pressures in type 2 diabetes patients with decompensated congestive heart failure

**DOI:** 10.1186/s13098-015-0116-2

**Published:** 2016-01-12

**Authors:** David Nathanson, Mats Frick, Bengt Ullman, Thomas Nyström

**Affiliations:** Department of Clinical Science and Education, Karolinska Institutet, 11883 Stockholm, Sweden; Department of Endocrinology and Diabetology, Södersjukhuset, 11883 Stockholm, Sweden; Department of Cardiology, Södersjukhuset, 11883 Stockholm, Sweden

**Keywords:** Atrial natriuretic peptide, Exenatide, GLP-1, Heart failure, Type 2 diabetes

## Abstract

**Background:**

The vascular effects exerted by GLP-1 are mediated by several synergistic mechanisms such as involvement of nitric oxide and natriuresis. Recently, it was demonstrated that atrial natriuretic peptide (ANP) is essential for the glucagon-like peptide-1 (GLP-1)-stimulated vascular smooth muscle relaxation that mediates anti-hypertensive action in rodents. Therefore a GLP-1–ANP axis has been suggested. The aim of this study was to investigate whether this effect can be demonstrated in patients with type 2 diabetes and congestive heart failure.

**Methods:**

The study was a post hoc analysis of a randomized double-blinded, placebo-controlled trial. Twenty male patients with type 2 diabetes and congestive heart failure were randomized to receive a 6-h infusion of exenatide or placebo. Cardiac filling pressures were measured by right heart catheterization, and plasma levels of ANP, N-terminal pro-brain natriuretic peptide, and exenatide were measured at baseline and at the end of the exenatide infusion.

**Results:**

Exenatide infusion resulted in a significant decrease of circulating ANP levels compared with placebo, concomitant with a decrease in pulmonary capillary wedge pressure (PCWP), pulmonary artery pressure (PAP) and right arterial pressure (RAP), and increased cardiac output. There was no correlation between plasma ANP levels and exenatide levels. A negative correlation between ANP levels and PCWP, PAP, and RAP, which remained significant after adjustment for plasma exenatide levels, was demonstrated during exenatide infusion.

**Conclusions:**

Exenatide infusion decreases cardiac filling pressure and ANP levels. The reduction of ANP levels was primarily because of the reduction in cardiac filling pressure, independent of exenatide levels. It seems unlikely that this was mediated via ANP.

Trial registration: http://www.isrctn.org/ISRCTN47533126

## Background

Glucagon-like peptide-1 (GLP-1) is a gut-derived hormone released after meal ingestion [1]. Beyond its well-known anti-hyperglycemic actions, studies have also shown beneficial effects on the vasculature [2, 3] and heart function [4–6]. Lowering of blood pressure has been observed in subjects with type 2 diabetes receiving long-term treatment with GLP-1 receptor (GLP-1R) agonists, although the mechanisms underlying this are not fully understood [7].

The vascular effects exerted by GLP-1 are mediated by several synergistic mechanisms as earlier studies have linked GLP-1 to both nitric oxide (NO)-dependent, and NO-independent vasorelaxation [3, 8, 9]. Additionally, antihypertensive effects of GLP-1 also might occur due to stimulation of natriuresis [10, 11]. Interestingly, it was demonstrated recently in rodents that atrial natriuretic peptide (ANP) might be essential for GLP-1-stimulated vascular smooth muscle relaxation, mediating anti-hypertensive actions. After activation of GLP-1R in cardiomyocytes with the GLP-1 agonists liraglutide and exenatide, an increase in cyclic adenosine monophosphate promoted membrane protein translocation, resulting in ANP release. This in turn induced cyclic guanosine monophosphate-mediated smooth muscle relaxation and natriuresis, and consequently blood-pressure reduction [12]. These findings prompted the investigators to define a novel GLP-1R–ANP axis, shedding light on understanding of the mechanisms of GLP-1-induced cardiovascular actions [13].

ANP is synthesized and stored in secretory granules of atrial myocytes [14, 15]. In patients with congestive heart failure (CHF), circulating ANP is associated with functional impairment assessed by the New York Heart Association (NYHA) classification, as well as elevated atrial pressure and other parameters of CHF [16–18]. A recent study has shown significant correlations between increases in plasma natriuretic peptides and GLP-1R-agonist-induced body composition changes [19]. Despite this, there is today no robust evidence for a GLP-1R–ANP axis in humans [10, 20, 21]. To the best of our knowledge, there are no studies investigating the association between GLP-1R and natriuretic peptides in patients with type 2 diabetes and CHF.

The current study is a post hoc analysis of a recently randomized controlled clinical trial, where the aim was to determine whether exenatide improves hemodynamic function in patients with type 2 diabetes and CHF [22]. Since a GLP-1R–ANP axis has been suggested in rodents [12], we aimed to investigate whether such an association between GLP-1R mediated hemodynamic effects and plasma ANP changes in patients with decompensated congestive heart failure, may take part. Therefore in the present study we retrospectively analyzed plasma levels for ANP and N-terminal pro-brain natriuretic peptide (NT-proBNP) on the one hand, and hemodynamic parameters, i.e. cardiac index (CI), pulmonary capillary wedge pressure (PCWP), pulmonary artery pressure (PAP), right arterial pressure (RAP), and peripheral resistance, on the other hand, following 6-h exenatide infusion.

## Methods

### Study population

The study population comprised 20 participants with type 2 diabetes hospitalized for CHF. Inclusion and exclusion criteria have been described elsewhere [22]. Briefly, patients were recruited from the Stockholm South Hospital, Sweden. Inclusion criteria were: male and female sex, age 18–80 years, known type 2 diabetes, hospitalization for CHF according to NYHA III-IV criteria, left ventricular (LV) systolic dysfunction with a documented LV ejection fraction of ≤35 % (assessed by echocardiography), and clinically stable period of 24 h using established therapy [diuretics, angiotensin-converting enzyme (ACE) or angiotensin-II inhibitors (A-II) inhibitors, and β-blockers]. Exclusion criteria were: type 1 diabetes, ongoing treatment with inotropic agents, acute coronary syndrome or documented acute myocardial infarction within the previous 8 weeks, active myocarditis, significant aortic stenosis or mitral/tricuspidal regurgitation, symptomatic primary pulmonary disease, ventricular arrhythmias, second-, or third-degree atrioventricular block, implanted cardioverter defibrillator or biventricular pacemaker, supine systolic blood pressure <85 or >200 mmHg, primary renal or hepatic impairment [estimated glomerular filtration rate (eGFR) <30 mL/min, aspartate aminotransferase/alanine aminotransferase >2 times the upper limit of normal], hypokalemia (<3.5 mmol/L) or hyperkalemia (>5.5 mmol/L), significant anemia (hemoglobin <100 g/L), pregnancy, or current/previous treatment with a GLP-1 receptor agonist or dipeptidyl peptidase-4 inhibitor.

The protocol was approved by the Swedish Central Ethical Review Board and the Medical Products Agency and conducted according to the principles of the Declaration of Helsinki, 1975. Written informed consent was obtained from all participants. The trial was registered at http://www.isrctn.org/ISRCTN47533126.

### Protocol

The trial used a randomized, crossover, double-blind design [22]. In summary the study protocol was performed in two sessions over 2 consecutive days. After an overnight fast, all participants underwent intravenous infusion with glucose (50 mg/mL; 50 mL/h) and insulin [Actrapid, Novo Nordisk, Bagsværd, Denmark, 1–6 U/h to maintain normoglycemia (4–6 mmol/L)], and exenatide (0.12 pmol/kg/min) or placebo, provided by Eli Lilly Amylin Alliance (Indianapolis, IN, USA) in a syringe pump device (Ivac Medical Systems, Basingstoke, UK) for 6 h. This was followed by an 18-h washout period. The placebo was the solvent used in the exenatide infusion. In the two-session block-randomization procedure, ten patients received exenatide on day 1 (Group A) followed by placebo on day 2. The remaining 10 patients received placebo on day 1 (Group B) followed by exenatide on day 2. Hemodynamic measurements were recorded at baseline, 1, and 6 h after the start of infusion. Blood samples for determining plasma ANP, NT-proBNP and exenatide levels were collected before the start of infusion (baseline) and after 6 h of infusion.

### Assessment of cardiac hemodynamics

Hemodynamic measures were determined by right-heart catheterization (method described elsewhere [22]). Thermodilution catheters were inserted via the internal jugular veins. The tip of the catheter was advanced into the pulmonary artery to reach a position adequate for monitoring wedge pressure. Then, 7.5-F pulmonary artery thermodilution catheters (AH-05050, Arrow International, Inc., Bernville, PA, USA), and the Siemens Sirecust SC 9000XL monitor (Siemens, Denver, CO, USA) were used to calculate cardiac filling pressure and CO.

### Invasive arterial blood pressure and heart rate measurements

A catheter with an arterial line primed with NaCl (0.9 %) was positioned in the radial artery in the right wrist of all patients. Calculation of HR was based on the average R–R interval over the final 10 s (Siemens Sirecust SC 9000XL).

### Biochemical analyses

ANP was determined by radioimmunoassay specific for h-ANP (EURIA-ANP, Euro Diagnostica, Malmö, Sweden). Exenatide was measured by sandwich immunoassay (Tandem Labs, San Diego, CA, USA). Two monoclonal antibodies (capture antibody and detection antibody, Tandem Labs, San Diego, CA, USA) were used to immobilize and detect exenatide.

NT-proBNP was determined by a sandwich immunoassay (Roche Diagnostics Scandinavia AB). Two monoclonal antibodies in a two-step procedure (capture antibody and detection antibody) were used to immobilize and detect brain natriuretic peptide (BNP). NEFA levels were determined using a NEFA-HR kit (Wako Chemicals, Neuss, Germany) on a Thermo T20xti instrument (Kone, Espoo, Finland).

### Statistical analyses

Data are presented as mean (±standard error of the mean) or percentages. Normal distribution of the variables was tested with the Shapiro–Wilk test. Differences in paired data were evaluated using the Wilcoxon signed-rank test. Correlations between hemodynamic variables and ANP were tested by Spearman’s correlation coefficients. To detect potential carry-over effects, Wilcoxon signed-rank tests between baseline levels for days 1 and 2 (for each variable studied) in Group A (the group that received active treatment on day 1) were performed. A general linear mixed model with participant and time as repeated factors and treatment as a fixed factor was used to test the effect of treatment on continuous parameters with data for more than two time points. To test whether the associations between ANP and NT-proBNP, and the hemodynamic variables were confounded by exenatide concentrations, we created multivariable linear regression models with ANP and NT-proBNP, respectively, as the dependent variable, and exenatide concentrations as a covariate. The assumptions for the linear regression models were tested as the standardized residuals were plotted against standardized predicted values to test statistical independence and variance of the errors. All tests were two-tailed, and a *P* value <0.05 indicated statistical significance. Statistical analyses were performed using the statistical software package SPSS 22.0 (IBM Corp., Armonk, NY, USA).

## Results

### Baseline characteristics

Baseline characteristics of the study population are shown in Table 1. Twenty male patients with baseline hemodynamic variables consistent with CHF with a depression of CO and an elevation of PCWP were included in the study.

**Table 1:** Participant characteristics

Parameter	Value
Patients (*n*)	20
Male/female	20/0
Age (years)	66 ± 1
BMI (kg/m^2^)	31 ± 1
Diabetes duration (years)	13 ± 2
Microalbuminuria (20–200 µg/min)	7/20
Mean (µg/min)	36 ± 18
Macroalbuminuria (>200 µg/min)	5/20
Mean (µg/min)	368 ± 63
Diabetic retinopathy	16/20
None	4
Mild non-proliferative	7
Moderate non-proliferative	7
Severe non-proliferative	2
HbA_1c_ (mmol/mol)	65 ± 4
Cholesterol (mmol/l)	4.1 ± 0.3
HDL-cholesterol (mmol/l)	1.0 ± 0.1
LDL-cholesterol (mmol/l)	2.4 ± 0.2
Triacylglycerol (mmol/l)	1.4 ± 0.1
eGFR (mL/min/1.73 m^2^)	64 ± 7
NYHA functional class (%)	
III	55
IV	45
LV ejection fraction (%)	26 ± 2
Risk factors for heart failure (%)	
CAD	60
Hypertension	80
DCM	10
Smoking (%)	
Former smoker	60
Current smoker	40
AF (%)	55
Concomitant medication (%)	
ACEi/A-II receptor antagonists	100
Beta-blocker	95
Loop diuretic	100
Spironolactone	40
ASA/clopidogrel	55
Warfarin	55
Diabetes treatment (%)	
Insulin	60
Metformin	25
Sulphonylurea	5
Combination therapy	5
Diet only	15

Values are mean ± standard error of the mean or proportions

*A-II receptor antagonists* angiotensin-II receptor antagonists, *ACEi* angiotensin-converting enzyme inhibitor, *ASA* aspirin, *AF* atrial fibrillation, *BMI* body mass index, *CAD* coronary artery disease, *DCM* dilated cardiomyopathy, *eGFR* estimated glomerular filtration rate, *HbA1c* glycosylated hemoglobin, *HDL* high-density lipoprotein, *LDL* low-density lipoprotein, *LV* left ventricular, *CAD* coronary artery disease, *NYHA* New York Heart Association

### Concomitant medications

Table 1 shows all medications used by study participants. All patients were on stable doses of ACE or A-II inhibitors, β-blockers (except one patient), and diuretics. All patients but three received antidiabetic therapy (12 received insulin, five received metformin and one received a sulfonylurea).

### Carry-over effects

We found no significant carry-over effects in any of the primary endpoint variables.

### Hemodynamic and metabolic parameters

The results of the original study including all hemodynamic data have been published elsewhere [22]. Hemodynamic effects are shown in Table 2. Briefly, after 6 h exenatide infusion there was a statistically significant increase in CI (0.3 ± 0.07 L/min/m^2^) as a result of increased heart rate (8 ± 3 bpm), but with a concomitant decrease in PCWP (−2.2 ± 0.9 mmHg) and RAP (−0.85 ± 0.7 mmHg) [22]. Stroke volume and measurements of peripheral vascular tonus such as: systolic-, diastolic-, and mean arterial blood pressure, and systemic vascular resistance did not change significantly during the exenatide infusion, data shown elsewhere [22].

**Table 2:** Hemodynamic and metabolic parameters during the study

Time point	Baseline^a^	0 h		6 h		*P*
		Exenatide	Placebo	Exenatide	Placebo	
Hemodynamic						
CI (L/min)	1.8 ± 0.1	1.8 ± 0.1	1.8 ± 0.1	2.1 ± 0.1*	1.9 ± 0.1	0.003^b^
RAP (mmHg)	9.0 ± 1	7.5 ± 1	8.5 ± 1	6.6 ± 1	7.9 ± 1	0.03^b^
PAP (mmHg)	28 ± 2	26 ± 2	28 ± 2	26 ± 3	29 ± 2	0.08 ^b^
PCWP (mmHg)	17 ± 2	14.8 ± 2	16.0 ± 2	12.6 ± 2^***^	17.4 ± 2	0.001^b^
Metabolic						
Exenatide (pmol/l)	0.6 ± 0.5	3.5 ± 3	1.9 ± 1	132 ± 11***	1.0 ± 0.5	0.001^b^
Glucose (mmol/l)	6.6 ± 0.3	6.8 ± 0.3	7.0 ± 0.3	6.0 ± 0.3	6.0 ± 0.2	0.2^b^
ANP (ρg/mL)	91.1 ± 13.3	97.1 ± 12.2	83.7 ± 13.8	78.5 ± 11.1	95.3 ± 12.7*	0.04^c^
ΔANP (ρg/mL)	–	–	–	−21.9 ± 9.2	12.5 ± 10.0*	0.02^c^
NT-proBNP (ng/L)	3793 ± 831	3954 ± 833	3919 ± 991	4262 ± 859	4519 ± 1182	0.4^c^
ΔNT-proBNP (ng/L)	–	–	–	307 ± 127	600 ± 249	0.5^c^
NEFA (mmol/L)	0.3 ± 0.06	0.3 ± 0.1	0.3 ± 0.1	0.6 ± 0.1*	0.5 ± 0.05	0.03^c^

Values are mean ± standard error of the mean

*CI* cardiac index, *RAP* right atrial pressure, *PAP* mean pulmonary arterial pressure, *PCWP* pulmonary capillary wedge pressure, *ANP* atrial natriuretic peptide, *ΔANP* changes in ANP levels following 6 h of infusion, *NT-pro-BNP* N-terminal pro-brain natriuretic peptide, *ΔNT-proBNP* changes in ΔNT-proBNP levels following 6 h of infusion, *NEFA* non-esterified fatty acids

* *p* < 0.05, ** *p* < 0.01, *** *p* < 0.001 for exenatide vs placebo

^a^Hemodynamic and metabolic parameters prior to the protocol (before any infusions)

*p* ^*b*^; overall *p* value for exenatide treatment vs. placebo over all time points (1, 3, 6 h) for every parameter, included in the model (general linear mixed model)

*p* ^*c*^; *p* value for exenatide treatment vs. Placebo after 6-h infusion (Wilcoxon signed-rank tests)

### Plasma levels of ANP, NT-proBNP and non-esterified free fatty acids (NEFA), and the correlation with exenatide plasma levels

**Fig. 1:**
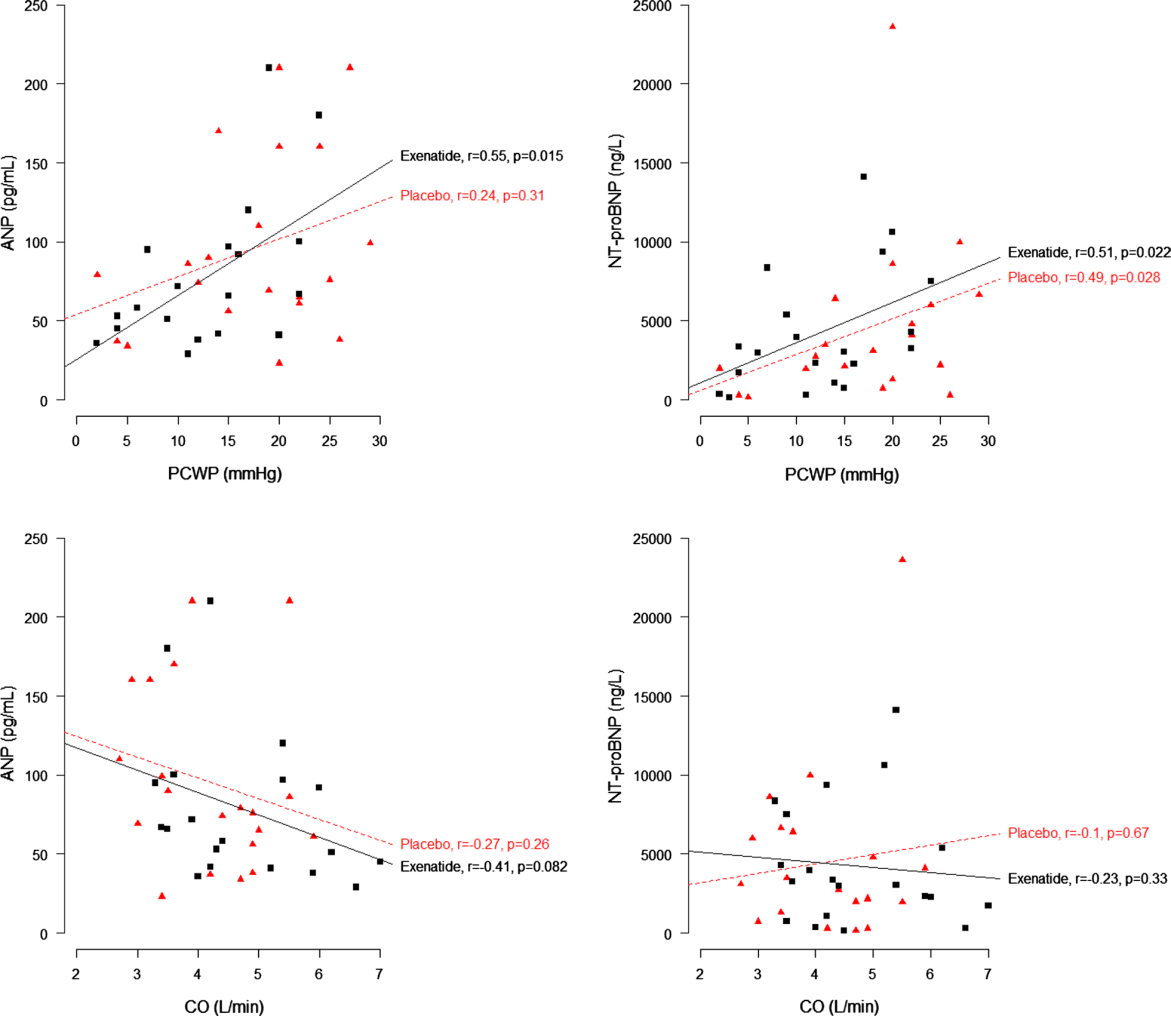
Correlations between ANP, NT-proBNP, exenatide concentrations and hemodynamic parameters after 6 h of exenatide infusion

**Fig. 2:**
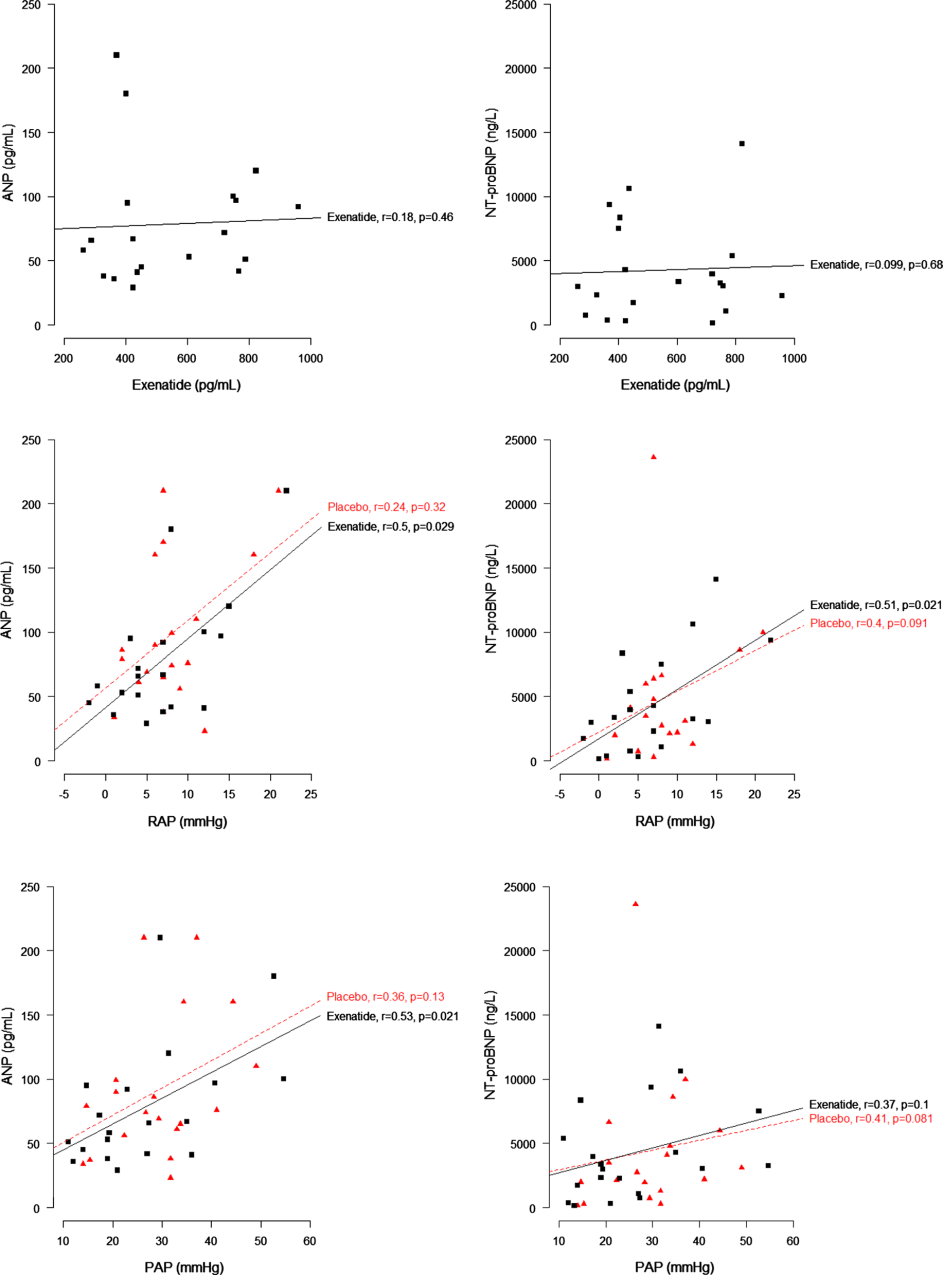
Correlations between ANP, NT-proBNP, exenatide concentrations and hemodynamic parameters after 6 h of exenatide infusion

As expected, plasma exenatide levels increased during the 6 h of exenatide infusion (0.6 ± 0.5 to 132 ± 11 pmol/mL) compared with the placebo infusion (0.6 ± 0.5 to 1.0 ± 0.5 pmol/L), *p* < 0.001. Exenatide infusion significantly decreased circulating ANP levels by 21.9 ± 9.2 pmol/mL (Table 2) compared with an increase during placebo infusion of 12.5 ± 10 pmol/mL (*p* = 0.02). Exenatide did not change NT-proBNP levels (*p* = 0.4; Table 2). Plasma levels of exenatide did not correlate with ANP levels (*r* = 0.18, *p* = 0.46), or NT-proBNP levels (*r* = 0.10, *p* = 0.68,) (Figs. 1, 2).

### Correlations between ANP, NT-proBNP and hemodynamic parameters during exenatide versus placebo infusion

There were significant positive correlations between ANP and RAP (*r* = 0.50, *p* = 0.029), PAP (*r* = 0.53, *p* = 0.021), and PCWP (*r* = 0.55, *p* = 0.015) during exenatide infusion, with no such correlations during placebo infusion (Figs. 1, 2). All correlations remained significant in linear regression models after adjustment for plasma exenatide levels (Table 3). There were significant positive correlations between NT-proBNP levels, PAP and PCWP during both exenatide and placebo infusions, with no such correlation for RAP (Figs. 1, 2). During exenatide infusion, ANP levels showed no correlation with NT-proBNP levels or CO (Figs. 1, 2).

**Table 3:** Multiple linear regression models with ANP as a dependent variable with adjustment for exenatide concentration

Variable	ß (95 % CI)	*P*	Adjusted R^2^
PAP	2.0 (0.3, 3.8)	0.025	0.19
PCWP	4.0 (0.8, 7.3)	0.017	0.22
RAP	5.6 (2.3, 8.9)	0.002	0.38

*ß* unstandardized regression-coefficient adjusted for exenatide concentration, *PAP* mean pulmonary arterial pressure, *PCWP* pulmonary capillary wedge pressure, *RAP* right atrial pressure

## Discussion

In the present study we found that 6-h exenatide infusion resulted in a significant decrease in ANP levels compared with placebo. No such effect was demonstrated for NT-proBNP levels. There was no correlation between ANP level and exenatide level. During exenatide infusion there was a significant positive correlation between ANP levels and the following hemodynamic variables: RAP, PAP and PCWP, which remained significant after adjustment for plasma exenatide concentration. This suggests that ANP levels were not directly associated with exenatide levels, but were related to the action of the decreased filling pressure during exenatide infusion.

In our previous study, we demonstrated that exenatide infusion increased cardiac index as a result of chronotropy, without any changes in stroke volume. However, favorable effects on cardiac filling pressures were also demonstrated, whereby RAP and PCWP significantly decreased [22]. As GLP-1 exerts pleiotropic actions on the cardiovascular system there may be several plausible explanations for our findings. GLP-1 and its analogues ameliorate cardiac dysfunction in several animal models of induced heart failure [23]. Some of these studies have suggested an increase in glucose use instead of lipid oxidation, and therefore more efficient utilization of the substrate [24], an issue that was beyond the scoop in the present study. However, as expected plasma glucose remained unchanged during exenatide infusion, as the study subjects received insulin-glucose infusion to achieve normoglycemia during the study protocol. Additionally, both vasodilatation and changes in renal hemodynamics could have decreased cardiac filling pressure: factors we were not able to control for. Even though it is established that GLP-1 exerts pleiotropic actions in the cardiovascular system it might, in the present study, be difficult to extrapolate blood pressure effects to those on vascular smooth muscle and vice versa due to heart rate as one confounding factor. Circulating ANP has been suggested as an important mediator to explain some of the beneficial cardiovascular actions of GLP-1 and its analogs [12].

It is well-known that plasma ANP is elevated in subjects with heart failure [25]. Atrial pressure is one major determinant for the release of ANP, because circulating plasma ANP levels are rapidly decreased when atrial pressure is reduced [26]. The ability to respond quickly to changes in cardiac filling pressures is maintained in patients with severe CHF [26]. Some parallel to this can be drawn from the present study. Our patients were to some extent adequately pharmacologically treated, as their cardiac filling pressure was only moderately raised, i.e., prior to randomization all patients had acutely received intravenous diuretics because of cardiac decompensation. There was a rapid decrease in ANP levels during exenatide infusion, which, together with reduced cardiac filling pressure, may suggest a correlation between plasma exenatide levels. However, no such correlation was demonstrated, but a significant correlation between ANP levels and cardiac filling pressure was observed. This finding merely reflects the reduction of atrial stretch [27, 28], rather than changes in ANP levels.

Both ANP and BNP levels are increased in patients with CHF. However BNP is a superior predictor of the severity of CHF [16–18, 29–31]. In the current study, NT-proBNP levels were strongly increased, indicating severe CHF. There were no changes in NT-proBNP levels during exenatide infusion. In contrast, there was a positive correlation between PCWP and NT-proBNP levels, regardless of whether exenatide or placebo was infused. BNP is synthesized primarily from the ventricles of the heart, and to a smaller degree by the atrium. Additionally, different patterns in secretions of ANP and BNP after volume loading/or pressor enhancements have been demonstrated [26, 32]. In such conditions, while circulating ANP levels increase rapidly, changes in BNP levels are negligible, supporting the fact that the atrium contains small amounts of BNP [26, 32]. In present study there was a rapid decrease in PCWP concomitant with a rapid decrease in ANP levels during exenatide infusion, with no such changes in BNP levels, which is supported by other studies [26, 32]. This may, in part, explain why the correlation between BNP levels and PCWP did not change between exenatide and placebo infusion.

There was no correlation between plasma exenatide levels and ANP levels during exenatide infusion, consistent with other recent studies in humans. These studies have shown that GLP-1 does not mediate ANP release [10, 20, 21]. However, this is in contrast to a recent murine study from Drucker and colleagues. In their study, the authors clearly demonstrated that the GLP-1 analog liraglutide mediated the release of circulating ANP, and led to a significant reduction of blood pressure because of vasorelaxation and natriuresis [12]. Because this finding could not be repeated in humans [20, 21, 25], the GLP-1–ANP axis might be species-specific. However, one recent study investigated type 2 diabetic obese patients, and demonstrated significant correlations in plasma ANP and BNP levels, and changes in liraglutide-induced weight loss [19]. As ANP and BNP can resist body-fat accumulation via increasing adipocyte lipolysis, the authors hypothesized that liraglutide-induced weight loss might be mediated by changes in circulating ANP and BNP levels [19], and not directly by GLP-1R. Although it has to be emphasized that the present study involved type 2 diabetes subjects with decompensated heart failure, and consequently these results cannot be generalized into a population without heart failure.

One marker of lipolysis is NEFA. In the current study, NEFA plasma levels were increased, both during exenatide and placebo infusion, compared with baseline. During exenatide infusion, NEFA levels were further increased without any changes in other metabolic parameters, i.e., glucose, insulin, C-peptide, glucagon and lactate levels. Importantly there was no correlation between plasma exenatide levels and ANP levels. With a lack of changes in metabolic parameters and no correlation observed between exenatide and ANP levels, it is more likely that factors such as tachycardia, i.e., reflecting a hyperadrenergic state, underlies the increased NEFA levels observed during exenatide infusion. Interestingly, ANP-induced lipolysis was shown to be species-specific, an effect that only occurs in primate fat cells [33]. This finding considers that other ANP effects, such as the GLP-R–ANP axis, also might be species-specific [12].

One strength of our study is the double-blinded, placebo-controlled, cross-over design. In addition, the invasive pulmonary artery catheter method remains the gold standard for monitoring cardiac filling pressure.

Several limitations of the study also must be noted. This study was not originally designed to investigate the effects of exenatide on neuropeptides, and the analyses of ANP and BNP were performed post hoc. ANP (and BNP) have natriuretic effects. In the current study, the decrease in filling pressure, evoked by exenatide, might simply be the result of increased natriuresis [10, 21], or even the indirect effect of the release of ANP [12]. We did not control for natriuresis. However, others have clearly demonstrated that the natriuretic effects evoked by GLP-1 [21] or liraglutide [10] are not mediated via ANP secretion. Finally, as this study was a cross-sectional, we cannot draw any conclusions regarding the pathophysiological significance of our findings.

## Conclusions

Our study demonstrates that short-term exenatide infusion resulted in a significant decrease in circulating ANP levels. There was no correlation between ANP level and exenatide level. During exenatide infusion there was a significant positive correlation between ANP levels and cardiac filling pressure, independent of exenatide levels.

## Abbreviations

ANP: atrial natriuretic peptide
BNP: brain natriuretic peptide
CHF: congestive heart failure
CO: cardiac output
GLP-1R: GLP-1 receptor
GLP-1: glucagon-like peptide-1
LV: left ventricular
NT-proBNP: N-terminal pro-brain natriuretic peptide
NYHA: New York Heart Association
NEFA: non-esterified free fatty acids
PAP: pulmonary artery pressure
PCWP: pulmonary capillary wedge pressure
RAP: right arterial pressure
